# Monocyte and Macrophage Functions in Oncogenic Viral Infections

**DOI:** 10.3390/v16101612

**Published:** 2024-10-15

**Authors:** Juliana Echevarria-Lima, Ramona Moles

**Affiliations:** 1 Laboratório de Imunologia Básica e Aplicada, Department of Immunology, Instituto de Microbiologia Paulo de Góes, Universidade Federal do Rio de Janeiro (UFRJ), Rio de Janeiro 21941-902, Brazil; juechevarria@micro.ufrj.br; 2 Department of Cell and Molecular Biology, University of Mississippi Medical Center, Jackson, MS 39216, USA; 3 Cancer Center and Research Institute, University of Mississippi Medical Center, Jackson, MS 39216, USA; 4 Center for Immunology and Microbial Research, University of Mississippi Medical Center, Jackson, MS 39216, USA

**Keywords:** monocytes, macrophages, viral infections, cancer

## Abstract

Monocytes and macrophages are part of innate immunity and constitute the first line of defense against pathogens. Bone marrow-derived monocytes circulate in the bloodstream for one to three days and then typically migrate into tissues, where they differentiate into macrophages. Circulatory monocytes represent 5% of the nucleated cells in normal adult blood. Following differentiation, macrophages are distributed into various tissues and organs to take residence and maintain body homeostasis. Emerging evidence has highlighted the critical role of monocytes/macrophages in oncogenic viral infections, mainly their crucial functions in viral persistence and disease progression. These findings open opportunities to target innate immunity in the context of oncogenic viruses and to explore their potential as immunotherapies.

## 1. Introduction

Intracellular pathogens, like viruses, need to enter permissive cells to replicate their genomes and avoid immune clearance. In chronic or persistent infections, the viral reservoir, a term referring to a site or cell type where the virus can persist and potentially reactivate, must have (1) a sufficient lifespan, (2) evade apoptosis, (3) interact with other cell types, and (4) escape specific immune responses. Some cell types have these characteristics constitutively; others acquire them following viral infections [1].

**Figure 1:**
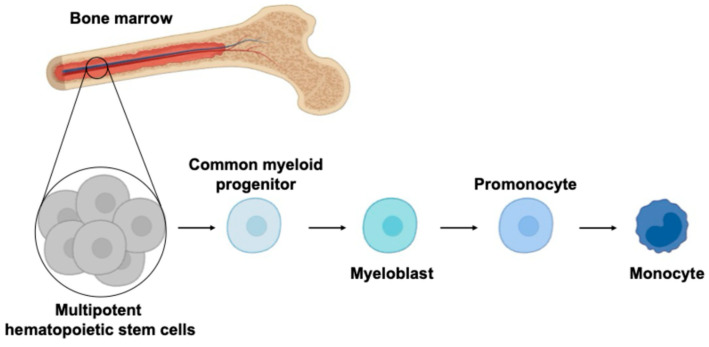
The figure represents monopoiesis, a process that leads to monocyte production from hematopoietic progenitors. The multipotent hematopoietic stem cells (HSCs) in the bone marrow give rise to the myeloid lineages. The common myeloid progenitor differentiates into myeloblasts and further to promonocytes, leading to production of mature monocytes that enter the systemic circulation.

**Figure 2:**
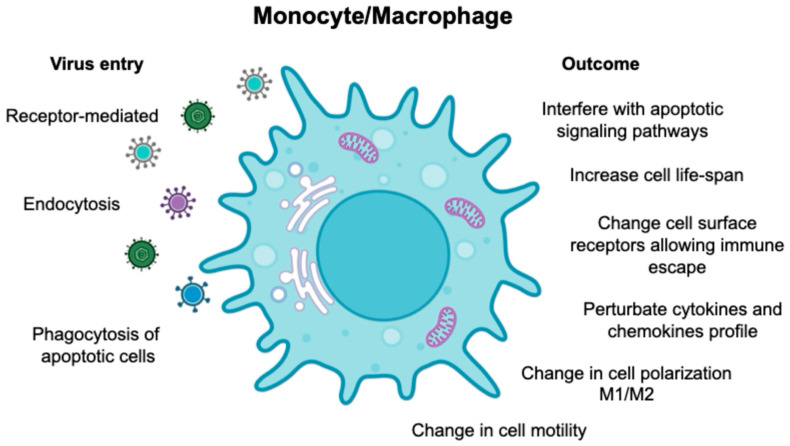
Schematic representation of viruses’ entry via receptors, endocytosis, and engulfment of apoptotic-infected cells. The figure also describes the biological events following the viral entry into monocytes and macrophages.

Monocytes, despite being non-dividing and short-lived, are unexpectedly targeted by different viruses, including human oncoviruses. This intriguing phenomenon suggests that viruses have evolved complex mechanisms to adapt monocytes as a reservoir for viral replication. Monocytes differentiate from bone marrow precursors and enter the systemic circulation (Figure 1), where as a part of the first line of defense, they interact with pathogens and other components of the immune system. Since monocytes possess a diverse array of phagocytic receptors, viruses can exploit these to establish infection [1]. The biology of monocytes is deeply affected by viral infection (Figure 2), causing the release of cytokines/chemokines, differentiation into long-lived macrophages, and migration into the tissues where they become resident-infected cells. Based on the Trojan horse hypothesis [2,3], viruses use this strategy to disseminate into different body compartments, creating a long-living viral reservoir and allowing virus replication.

**Figure 3:**
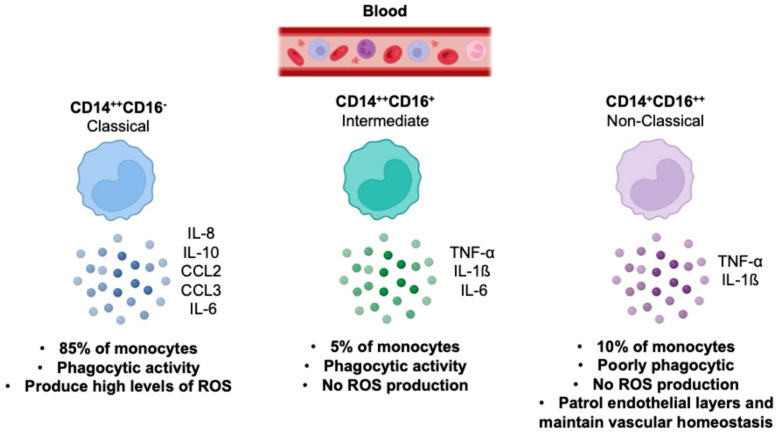
The illustration describes the main characteristics of classical, non-classical, and intermediate monocyte subsets in human health [6].

Based on the expression of surface markers, monocytes are divided into three subsets: classical (CD14++CD16−), non-classical (CD14+CD16++), and intermediate (CD14++CD16+). It is well established that each monocyte subset has differing abilities to secrete cytokines and respond to pathogen-associated molecular patterns (PAMPs). Classical monocytes, which account for ~85% of circulating monocytes, are phagocytic and secrete inflammatory cytokines. Conversely, the non-classical monocytes constitute ~10% of the total monocyte population, are poorly phagocytic, and secrete TNF-α in response to some stimuli but less of other pro-inflammatory molecules. Intermediate monocytes, which represent the remaining ~5%, are increased in diseases such as severe asthma, rheumatoid arthritis, and sarcoidosis [4,5] (Figure 3). Furthermore, all subsets produce cytokines that contribute to an inflammatory microenvironment that may foster inflammatory diseases and cancer progression [6]. This evidence underscores the pressing need to better understand monocyte/macrophage biology for the development of novel antiviral and cancer therapies.

**Figure 4:**
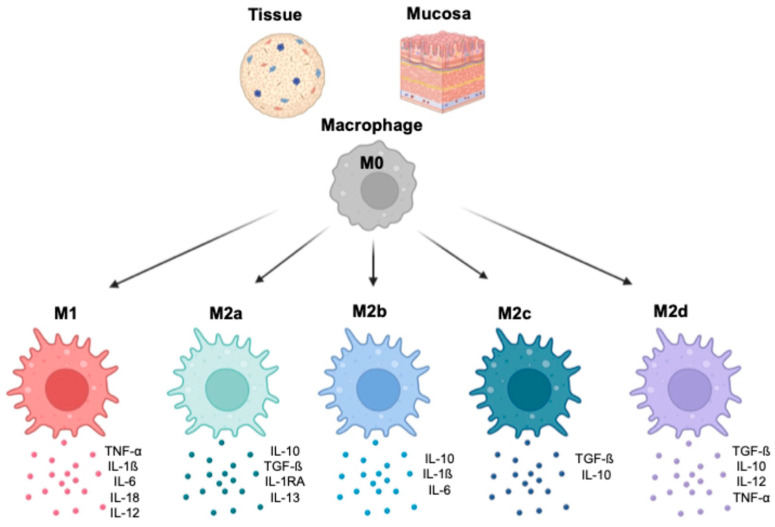
In the tissues and at the mucosa level, M0 macrophages polarize toward M1, M2a, M2b, M2c, and M2d. Each phenotype is characterized by exhibiting specialized functions and unique cytokine profiles (the most relevant cytokines are reported in the figures).

Monocytes migrate from the systemic circulation into tissue and/or mucosa, where they differentiate into macrophages. This process is characterized by the expression of specific surface markers. A subset of mucosal- and tissue-resident macrophages originates from their precursors, peripheral blood monocytes, which respond to chemotactic stimuli. Other subsets, such as liver Kupffer’s cells (KCs), populate their compartments during early fetal life, and their numbers are maintained by proliferation without requiring additional progenitors [7,8] (Figure 4).

Macrophages differentiate in response to a complex array of specific signals, including growth factors, cytokines, and cell-to-cell interactions [9,10,11,12]. This intricate process leads to the polarization toward M1 or M2 macrophages, which have two distinct phenotypic patterns and functional properties [13]. Polarized M1 macrophages are induced by pro-inflammatory cytokines, including interferon-γ (IFN-γ), tumor necrosis factor α (TNF-α), bacterial lipopolysaccharide (LPS), granulocyte–macrophage colony-stimulating factor (GM-CSF), and macrophage colony-stimulating factor (M-CSF) [14]. M1 macrophages typically release pro-inflammatory cytokines, such as IL-1β, IL-6, IL-12, IL-18, and TNF-α, [15,16,17]. IL-12 promotes Th1 differentiation, which is crucial for antiviral responses [18] (Figure 4). On the other hand, M2 macrophages play an essential role in maintaining immune balance by releasing anti-inflammatory cytokines, such as IL-10 and TGF-β, suppressing immune responses [19]. Four subsets of M2 macrophages have been identified based on specific functions, surface markers, and cytokine profiles (Figure 4). An important consideration is that the M1 and M2 classification is a simplification, and macrophages might display overlapping phenotypes depending on their tissue location and type of signaling.

Interestingly, macrophages can also derive from myeloid-derived suppressor cells (MDSCs), which is an immature myeloid cell population with immunosuppressive effects involved in chronic infections and cancer [20,21,22]. MDSCs originate in the bone marrow and later differentiate into granulocytes, mature dendritic cells, or macrophages. In different pathological conditions, MDSCs migrate and accumulate in tumors, spleen, bone marrow, and blood. MDSCs can be divided into monocytes, granulocytes, and endothelial-committed subsets based on their origins and surface markers. MDSCs suppress the function of other immune cells and create an immunosuppressive environment by inhibiting T-cell activation [23,24].

Monocytes could also differentiate into dendritic cells (DCs), which bridged innate and adaptive immune responses [25]. DCs are crucial in the clearance of viruses, by secreting cytokines, such as type I interferon (IFN-I), required to achieve viral clearance [26,27,28]. DCs are professional antigen-presenting cells (APCs). Following the uptake, antigens are processed and presented to naïve T lymphocytes, priming antigen-specific immune responses [29]. DCs express membrane and cytosolic receptors that recognize pathogens, cancer cells, and infected cells [30]. DC biology and functions are controlled by environmental signals, such as pathogen-associated molecular patterns, damage-associated molecular patterns, and cytokines, which are sensed by extracellular and intracellular receptors, including pattern recognition receptors (PRRs) [31]. DCs also play a crucial role in cancer, since they are a central component of the tumor microenvironment (TME) [32]. During inflammatory responses, monocytes migrate into the inflammatory site and differentiate into DCs named monocyte-derived DCs (moDCs) [32,33]. The chemotactic (C-C Motif) chemokine ligand 2, CCL2, released by tumor cells recruited monocytes into the TME [34]. Based on environmental signals, moDCs might possess an antitumor phenotype or differentiate into immunosuppressive cells, favoring cancer progression. Stromal factors, such as CCL2, IL-6, and TGF-β1, produced by tumor-associated stromal myofibroblasts induce an immunosuppressive phenotype by increasing the expression of CD209 and PD-L1. Upon lipopolysaccharide (LPS) stimulation, these moDCs release high levels of IL-10 and less IL-12. Moreover, they suppress T-cell proliferation and fail to cross-present tumor antigens to CD8+ T cells [35]. This phenotype favors cancer progression.

In conclusion, viruses have evolved complex mechanisms to manipulate and exploit monocytes and macrophages to disseminate throughout the body and establish chronic infections. This review delves into the published data on myeloid cell interactions during viral infections, with a primary focus on oncogenic viruses. Understanding these interactions is not only a fascinating scientific endeavor but also a crucial step toward developing effective therapeutical strategies for viral diseases.

### Monocytes and Macrophages in Cancer

The role of monocytes and macrophages in regulating inflammation is central in cancer initiation and progression. Mantovani and colleagues identify inflammation as the seventh hallmark of cancer, and infectious disease literature supports this hypothesis [36]. Experimental evidence demonstrates the importance of inflammation in creating a tumor-promoting microenvironment [37]. In myeloid cells, inflammation is regulated by the transcriptional factors [38,39] that activate the transcription of pro-inflammatory cytokines such as IL-12 and TNF-α. Inflammatory response is associated with cancer initiation [38]. Inhibition of inflammation in myeloid cells reduces tumor progression in vivo [40]. Chronic infection, induced by pathogens, is also associated with increased cancer risk [36], by activating macrophages which generate reactive nitrogen and oxygen species [41], creating a mutagenic environment [42].

Cancer is characterized by elevated peripheral blood monocyte counts [43,44,45], typically associated with a worse prognosis [46,47,48,49]. Elevated monocyte levels can be caused by enhanced mobilization from the bone marrow or increased monopoiesis. The central regulator of monocyte mobilization from the bone marrow is CCL2, which is often highly accumulated in the serum of cancer patients [50,51,52,53]. Additionally, emerging evidence suggests that tumors might influence hematopoiesis [44]. The relevance of monocytes in cancer is also associated with their ability to differentiate into tumor-associated macrophages (TAMs). TAMs support cancer progression through different mechanisms. They secrete growth factors to favor oncogenesis, which supports tumor growth. TAMs also produce proteolytic enzymes and motor-related proteins to enhance tumor invasion and metastasis. Finally, TAMs are crucial in creating an immunosuppressive microenvironment that favors cancer immune evasion [54,55]. Evidence suggests that classical monocytes might give rise to TAMs, which promote tumor progression. In prostate tumors, for example, blood monocyte counts correlate with increased tumor-infiltrating macrophage [49]. However, more studies are needed to confirm if this correlation is a general phenomenon.

Meta-analysis data also show a correlation between macrophage density and poor patient prognosis [56], which is regulated by growth factors and chemokines, such as colony stimulating factor 1 (CSF1), GM-CSF, IL-3 [57], and CCL2 [58], promoting growth, differentiation, and chemotaxis. Overexpression of CSF-1, the central lineage regulator for macrophages [58], is commonly associated with poor prognosis in cancer patients [36,59,60,61,62,63,64]. CCL2 is also over-expressed in a wide range of cancers [36] and is associated with poor prognosis [65,66,67]. Notably, in cervical cancer, its absence is associated with increased survival [68]. Overall, these studies demonstrate the involvement of macrophages in cancer initiation or progression.

In conclusion, oncogenic viruses affect monocyte and macrophage functions contributing to cancer initiation and progression.

## 2. Hepatitis B Virus (HBV)

Despite the prophylactic vaccine, HBV infection is a global health crisis, affecting 296 million people worldwide. Inadequate immune response to HBV infection results in chronic illness, leading to over 240 million cases of chronic HBV infection. This chronic condition puts patients at risk of developing virus-associated diseases, including liver cirrhosis and hepatocellular carcinoma, which claim the lives of 500,000 individuals annually [69,70,71,72,73].

HBV, a member of the Hepadnaviridae family, is a small DNA virus, which replicates through an RNA intermediate and integrates into the host genome. This unique replication cycle confers a distinct ability of the virus to persist in infected cells. HBV replication occurs in the hepatocytes as soon as the infection begins, resulting in high-rate virions and HBV protein secretion that might persist for decades. The persistence of HBV in the hepatocytes, even decades after infection, underscores the severity and longevity of this global health condition [74,75,76].

### 2.1. HBV and Monocytes

Research has shown that monocyte function is impaired in HBV infection [77]. The frequency of the different monocyte subsets is altered depending on the clinical phase of the disease [78]. The main viral protein, Hepatitis B surface Antigen, HBsAg, binds monocytes and suppresses their activity. Several studies have shown that HBsAg inhibits the release of LPS-induced cytokines in human monocytes by interfering with Toll-like receptor (TLR) signaling [79,80,81]. Specifically, HBsAg inhibits TLR2-induced phosphorylation of p38 MAPK and JNK MAPK, reducing the production of IL-6, TNF-α, and IL-12 [79]. Previous findings have also shown that HBsAg interacts with monocytes. This interaction induces MyD88–NF-κB signaling, leading to increased expression of the inhibitory molecules PD-L1, IL-10, and transforming growth factor beta (TGF-β), thereby initiating an immunosuppressive cascade [82]. The binding between HBsAg and monocytes is intensified by heat-labile serum protein and inhibited by Ca2+/Mg2+ low pH and HBsAg-specific monoclonal antibodies [83]. HBsAg not only inhibits monocytes’ inflammatory response but also regulates NK cell function, interfering with IFN-γ production by blocking IL-18 and IL-12 production. As the most abundant HBV protein in the liver and in the peripheral blood of chronic HBV-infected patients, HBsAg can accumulate up to 100 mg/mL, hampering immune responses [84].

HBsAg is not the only viral protein that regulates host immune responses in HBV infection. The hepatitis B e-Antigen (HBeAg), which is released from the infected hepatocytes into the systemic circulation, also suppresses inflammatory cytokines. In chronic HBV-infected individuals, the expression of TLR2 negatively correlates with HBeAg concentration. Stimulation with TLR2 agonist of peripheral blood mononuclear cells (PBMCs) isolated from patients positive for HBeAg produces less TNF-α and IL-6 than HBeAg-negative patients [85]. Moreover, exposure of monocytes to HBeAg suppressed LPS-inducing TNF-α and IL-1β both in vitro and in vivo [86,87]. Overall, these data demonstrated that HBV viral proteins severely impair monocyte functions by reducing proinflammatory cytokines, favoring viral immune escape.

### 2.2. HBV, Macrophages, and Kupffer Cells

The impact of HBV infection is not limited to monocytes; macrophages are also significantly affected, especially the hepatic macrophages, Kupffer cells (KCs). Macrophages participate in host defense against pathogens and interact with lymphocytes by activating or inhibiting surface molecules. Macrophages are particularly important in HBV infection, which is typically associated with T-cell dysfunction. HBV infection is characterized by the activation of anti-inflammatory macrophages with increased IL-10 production, which supports the functional inactivation of CD8+ T cells [88,89].

KCs are macrophages that represent 15% of the total liver and play a crucial role in HBV. KCs maintain liver homeostasis by ingesting and degrading cellular debris, foreign material, and pathogens [90,91,92]. Furthermore, KCs are involved in different immune responses, such as immune cell activation, antiviral response, and tissue damage repair [93]. KCs regulate T-cell responses and differentiation by using the costimulatory surface molecules CD80 and CD86 [94,95]. CD80 drives T-cell differentiation toward T helper type 1 (Th1), which promotes a proinflammatory response, while CD86 differentiates toward Th2, which induces an anti-inflammatory response. In HBV infection, T cells mainly initiate Th2 immune responses rather than Th1 [88,89], which inhibit antiviral responses favoring HBV persistence.

In HBV humanized mice, HBV induces monocytes/macrophages to differentiate into anti-inflammatory M2 macrophages, expressing immunosuppressive cytokines, such as IL-10 and TGF-β [96]. Similarly, in chronic HBV-infected patients, increased levels of immunomodulatory mediators, including IL-10 and TGF-β, and expression of PD-L1 and PD-L2 were observed in KCs, resulting in anti-HBV T-cell response suppression [82]. Mice models of HBV have also shown that upon HBV infection, elderly mice display more TNF-α-producing Ly6C+ monocytes and fewer IL-10-secreting KCs compared to younger mice, favoring HBV clearance [97].

KCs can have opposite roles in the presence of different HBV antigens [98]. Boltjes et al. demonstrate that KCs interact with HBsAg, inducing secretion of the IL-6 and TNF-α proinflammatory cytokines that were substantially increased compared to healthy controls [99]. Conversely, in vivo experiments have shown that HBcAg (core antigen) interacts with KCs following TLR2 activation, mediating humoral and cellular tolerance via IL-10 production. As expected, in vivo, TLR2 knockout or KC depletion improved HBV-specific CD8+ T-cell responses and accelerated HBV clearance [100]. On the other hand, the hepatitis B envelope antigen (HBeAg) inhibits IL-1β maturation by blocking NF-κB phosphorylation and reactive oxygen species (ROS) production [101]. Interestingly, HBeAg plays a dual role on macrophage function. In the HVB mice model, maternal HBeAg induces PD-L1 expression in KCs upon HBV infection, promoting their polarization toward M2 anti-inflammatory macrophages. In this context, M2-like KCs suppress the HBV-specific cytotoxic T-lymphocyte (CTL) response, supporting viral persistence. However, in control mice born from HBeAg-negative mothers, HBeAg promotes the M1 proinflammatory KCs macrophages favoring viral clearance [102].

In addition to KCs, monocyte-derived macrophages are also crucial in antiviral immune response and HBV pathogenesis. Intrahepatic macrophages that phagocytosed HBcAg display an anti-inflammatory phenotype, which favors the maintenance of infection. Moreover, HBcAg from infected hepatocytes, the virus’s primary target, enhances macrophage-mediated angiogenesis and upregulates IL-23 secretion in monocyte-derived macrophages [103]. In addition, HBV-induced M2-like hepatic macrophages promote the immunosuppressive activity of regulatory T (Treg) cells by enhancing cytotoxic T-lymphocyte-associated antigen 4 (CTLA-4) and CD39 expression in an amphiregulin-dependent manner [104]. Promoting Treg response impairs T helper type 1 (Th1) cell immune responses that are crucial in fighting viral infection and accelerates HBV liver fibrosis and pathogenesis.

In the context of HBV, DCs play a crucial role in chronic disease caused by the virus. There are no differences in the number of circulating DCs in HBV-infected patients [105,106]. However, DCs from HBV patients display a tolerogenic state characterized by impaired antigen-presenting capacities, decreased migration, and impaired cytokine production, favoring viral persistence [107,108,109].

## 3. Hepatitis C Virus (HCV)

HCV is a positive single-stranded RNA virus responsible for chronic diseases, including liver failure and hepatocellular carcinoma [110,111,112]. Following infection, HCV evades host immunity and establishes persistence leading to the development of chronic illness. Acquired immunity is essential in fighting and eradicating infection. On the other hand, innate immunity is crucial in shaping the adapting immune response by regulating T-cell differentiation and determining the infection outcome [113,114,115].

### 3.1. HCV and Monocytes

The tropism of HCV is mainly hepatocytes; however, several lines of evidence have shown that monocytes and macrophages are involved in HCV persistence and pathogenesis. In fact, monocytes are crucial in vivo for HCV replication, showing viral particle production. Interestingly, all the monocyte subsets CD14+CD16++, CD14++CD16++, and CD14+CD16− cells were found to be infected in patients [116,117], containing the core HCV protein (HCVc) [118]. Monocytes are not only infected by the virus; HCV protein expression also changes their cellular biology. In chronically infected HCV patients, monocytes have higher Galectin-9 (Gal-9) expression, which is a protein that regulates the interaction between monocytes and T cells [116]. Gal-9 binds to a receptor named T-cell immunoglobulin mucin domain 3 (Tim-3), which is persistently expressed on dysfunctional T cells during chronic infections [119]. This might be a cause of T-cell suppression typically observed in HCV-infected patients [116].

Additionally, monocytes isolated for HCV-infected individuals display perturbation of cytokine production, creating an immunological landscape that favors viral persistence and disease progression. Consistently, suppression of IL-12 production in chronically HCV-infected patients has been reported. IL-12 is a pro-inflammatory cytokine that plays a crucial role in T-cell activation and differentiation, and it also regulates the expression of Programmed Death 1 receptor (PD-1), an inhibitory surface receptor expressed by immune cells [73]. Notably, in HCV infection, monocytes and macrophages display decreased IL-12 production, which correlates with an increased PD-1 expression. Interestingly, resumed IL-12 production ex vivo is observed with PD-1/PD-L1 blocking antibodies and combination treatment with pegylated interferon plus ribavirin (INF/RBV). The possible mechanism is STAT-1-dependent since the treatment enhances its phosphorylation [120]. Other researchers, however, showed that the inhibition of IL-12 production in monocytes and macrophages is due to the negative regulator Tim-3 [121].

Monocytes are not only involved in supporting viral replication and cytokine production, but also able to differentiate into MDSCs, an immature myeloid population with immunosuppressive effects [20,21,22]. In chronic infection, MDSCs create a suppressive environment inversely correlated with T-cell frequency in the prereferral blood [23,24]. Monocytes stimulated with HCV core protein and poly I: C result in increased TNF-α (pro-inflammatory), IL-10 (anti-inflammatory), and INF-γ (antiviral) cytokine production. Interestingly, these cytokines are known to be involved in the monocytes’ reprogramming to acquire the MDSC phenotype. HCV induces differentiation of monocytes into MDSCs by activating the PI3K pathway via IL-10 and TNF-α. Monocytes play a significant role as the primary producers of IL-10 in HCV infection. The anti-inflammatory cytokine, IL-10, can decrease the frequency of CD8+ T cells and impair their differentiation. Consistently, in HCV-infected individuals, T-cell function is impaired. The elevated levels of IL-10 in the plasma of chronic hepatitis C (CHC) patients and their high degree of IL-10 reliance, both spontaneously and following HCV antigen stimulation [122], highlight its potential as a therapeutic target.

Collectively, these data highlight the significant role of monocytes in HCV infection. In addition, HCV impairs DC functions and evades the antiviral response by inhibiting IFN-I secretion. In the context of HCV-infected liver, DCs are highly present despite the infection’s potential to decrease their number. Cell-to-cell contact between HCV-infected cells and DCs affect IFN-I secretion, promoting virus immune evasion [123].

### 3.2. HCV and Macrophages

Not only monocytes are important in HCV-associated diseases, but also macrophages are important due to their long persistence in the liver. In patients, HCV was detected in liver macrophages up to 9 years following treatment [124]. Despite the tropism of HCV to hepatocytes, macrophages are crucial in in vivo replication, showing production of the virus as long as macrophages survive [125]. In addition to hepatocytes, microglial cells of HCV-infected individuals have been shown to be virus-positive, demonstrating significantly increased levels of IL-1α, IL-1β, TNF-α, IL-12, and IL-18 proinflammatory cytokines and increased transcription of chemokines IL-8, IL-16, and IP-10. Scientists in the field have speculated that the release of proinflammatory cytokines, neurotoxins (like NO), and HCV viral proteins upon infection could potentially affect brain function, leading to neurocognitive dysfunction and depression [126]. Macrophages following activation by HCV are involved in viral hepatic inflammation and fibrosis progression [127]. The current therapeutical approach for patients is direct-acting antiviral (DAA) therapy, which aims for viral clearance in hepatocytes. However, recent data showed that treating myeloid cells had a beneficial effect. The soluble CD163 (sCD163) is released from activated liver macrophages in chronic HCV patients. Successful treatment with DAA rapidly reduces sCD163 and is associated with decreased histological inflammatory activity and fibrosis, suggesting rapid macrophage deactivation upon HCV clearance. These data confirmed the relevance of macrophages in HCV liver-associated diseases and validated sCD163 as a marker of hepatic inflammatory activity and fibrosis [127].

Overall, the discussed data underscore the significant role of monocytes and macrophages in HCV infection. This discovery brings light into the understanding of the complex role of innate responses in HCV infection, opening opportunities for the development of future treatment.

## 4. Epstein–Barr Virus (EBV)

Epstein–Barr virus, EBV, the first human oncovirus to be identified, is a ubiquitous pathogen that infects most adults worldwide [128,129]. Its role in oncogenesis, particularly in diseases such as Burkitt’s lymphoma, nasopharyngeal carcinoma (NPC), and various tumors associated with immunosuppression, e.g., post-transplantation and AIDS, is of significant importance. B lymphocytes and epithelial cells are primary targets of the virus, although EBV can also infect other cell types [130,131,132]. However, the complexity of EBV’s interaction with the host is further highlighted by data showing that EBV infects and replicates in macrophages [133,134]. Human monocytic cell lines transformed by EBV display EBV nuclear antigen 1 (EBNA-1) and latent membrane protein 1 (LMP-1), but not EBV nuclear antigen 2 (EBNA-2). These cells were found to be tumorigenic following injection into nude mice [134]. Moreover, EBV was found to promote monocyte survival and maturation. For this reason, monocytes are possibly used by the virus as a vehicle of dissemination, a process that underscores the intricate nature of EBV’s pathogenesis [135].

By using tongue and buccal explants with EBV, Tugizov et al. demonstrated that EBV first infected submucosal monocytes, which then migrated into the epithelium and spread to oral epithelial cells. These cells are also known as EBV-positive macrophages or oral hairy leukoplakia tissues [136]. By using the genetically EBV-related, Murine gammaherpesvirus 68 (MHV68), Li et al. demonstrated that replication-defective MHV68 mutants establish long-term latent infection in macrophages, while latent infection in B cells seems to require a lytic infection [137]. Moreover, enhanced outgrowing of EBV-transformed chronic lymphocytic leukemia B cells is mediated by cocultivation with macrophage feeder cells [138].

Inflammation is a critical factor that induces carcinogenesis and tumor-associated macrophages (TAMs), which are immune cells heavily involved in cancer-related inflammation. TAMs enhance tumor progression and metastasis. In the context of EBV, TAMs have been detected in many EBV-associated neoplasms, a finding that underscores the significant role of macrophages in EBV’s pathogenesis. In situ hybridization with EBV antisense RNAs combined with anti-CD68 antibody staining shows that TAMs infiltrate EBV-associated neoplasms and they express EBV transcripts [139]. All of these data suggest a strong influence of macrophages in EBV oncogenesis.

The EBV protein BamHIC is 84% identical to human IL-10, a proliferation and activation factor of B cells, a major virus target. The impact of EBV IL-10 on multiple cell types, including T and NK cells, is central to the virus’ pathogenesis. Specifically, EBV IL-10 is believed to be important in establishing viral longevity and significantly suppressing host immune responses [140,141]. Therefore, understanding the function of EBV IL-10 is important in both virology and cancer biology. Endogenous IL-10 and EBV IL-10 strongly reduce antigen-specific T-cell proliferation by decreasing monocytes’ antigen-presenting capacity [142]. IL-10 and EBV IL-10 also promote monocyte differentiation to macrophages and block differentiation into dendritic cells. Transfection of EBV IL-10 into murine melanoma cells resulted in palpable tumors in 4 weeks and a local immunosuppressive effect, with reduced tumor infiltration of CD4+ and CD8+ T cells [143]. EBV infects T and B lymphocytes, Langerhans cells, epithelial cells, and macrophages. In Langerhans cells, epithelial cells, and T and B cells, EBV induces proliferation and cellular transformation. EBV IL-10 activates and differentiates monocytes into macrophages to develop TAMs, resulting in the progression of EBV-associated tumors. Furthermore, it has been shown that the EBV protein EBNA-1 activates the bone morphogenic protein (BMP) that, in turn, enhances tumor proliferation via IL-10-dependent M2 polarization of TAMs within renal cell carcinoma cell lines and patient samples [144,145]. Lee et al. showed that BMP/IL-10/CD68 was associated with poor prognosis, suggesting that EBV manipulates the macrophage compartment to promote oncogenesis and disease progression [144].

EBV not only infects B lymphocytes and epithelial cells, but also DCs [146]. The infection activates DCs, contributing to the innate restriction of EBV infection and initiating EBV-specific adaptive immune responses. DC activation makes B cells less prone to viral-induced transformation, partially restricting disease progression [147]. This environment will activate other innate cells supporting control and viral clearance [148].

## 5. Human Papillomavirus (HPV)

HPV is a small, non-enveloped, circular, double-stranded DNA virus that infects skin or mucosal cells. What makes HPV infection a serious condition is its potential to cause six types of cancer: oropharyngeal, anal, penile, vulvar, vaginal, and most notably, cervical cancer (CC) [149,150]. Constantly, persistent infection with high-risk HPV has been shown to cause abnormal cell growth, which may lead to cancer development. If left untreated, persistent infection of the cervix causes 95% of CC, which is the most common gynecological malignant tumor worldwide [151,152]. The main risk factor for the development of CC is the persistent infection of high-risk HPV-16 and -18, which is responsible for 80–90% of all cases [153]. Several lines of evidence have shown that persistent HPV-16/18 infection results in the integration of the HPV genome into the host genome, altering immune responses and accumulating microlesions that lead to the development of CC [152]. Recent studies have shown that HPV infection causes smoldering inflammatory responses and oxidative stress, which is essential in the development of CC [154,155]. Additionally, virus-induced epigenetic changes in the infected cells are involved in the evolution of HPV pathogenesis.

Inflammatory cells connect tumors and inflammation, which is a critical factor in oncogenesis and cancer progression. Tumors are commonly surrounded by many inflammatory cells, including dendritic cells, mast cells, and macrophages. These cell types interact with cancer and vascular endothelial cells, creating the tumor microenvironment (TME) [156]. The ‘seed and soil hypothesis’ is a concept in cancer biology that suggests a tumor (the ‘seed’) requires a suitable environment (the ‘soil’ or TME) for growth and progression [157]. Macrophages are crucial components and constitute 30 to 50% of TME; they are named tumor-associated macrophages (TAMs) for their function [37,158].

TAMs, as central components of the tumor microenvironment, play a pivotal role in the progression of CC associated with chronic HPV infection [159]. In the development of CC, TAMs and tumor angiogenesis are closely related. From chronic cervicitis to invasive CC, macrophages and new blood vessels in the tumor microenvironment increase synchronously. The number of TAMs in the cervical lesion matrix increases during the progression of CC. M1 macrophages release pro-inflammatory factors, which trigger an immune response that decreases the severity of CC. In the CC matrix, the polarization of macrophages is regulated by lactate, which is released by cancer cells [160]. This results in TAM polarization toward M2 macrophages, which correlates with poor prognosis and poor response to therapy [161]. M2 polarization induces increased surface expression of CD163 and IL-10 release. The expression of CD163 is a better predictor compared to CD68 of malignant transformation and the metastatic potential of CC [162]. Additionally, IL-10 is a key cytokine involved in CC evolution by regulating the transcription of immune cells, preventing the killing of cancer cells, and promoting the occurrence, development, and metastasis formation in CC [163,164,165].

DCs also play a significant role in cervical cancer induced by HPV [166]. Several studies have shown that DCs display an impaired phenotype in HPV infection, including lower numbers [167,168,169], changes in their morphological characteristics [170], and decreased capacities to activate immune responses [171,172]. Keratinocytes are the main target of HPV. However, DC subtype Langerhans cells (LCs) are critical in infection regulation [166,173,174]. Interestingly, HPV infection of LCs results in no viral gene expression and viral particle production [166,173]. Upon infection, keratinocytes secrete pro-inflammatory cytokines that result in LC recruitment and activation [175,176,177]. HPV recognition by DCs induces their activation and eventual maturation [166,174,176]. However, once the tumor is established, infected keratinocytes are also reported to release the anti-inflammatory cytokines TGF-β and IL-10, down-modulating the activation of LCs [177]. These events increased HPV capacity for immune evasion. DCs, especially LCs, play a significant role in HPV-induced cervical cancer [166]. Immature LCs sense the extracellular environment, uptaking and processing antigens, and migrate to the lymph nodes. Several studies showed that LCs exhibit an impaired phenotype in HPV infections [167,168,169,170,171,178]. Lower frequencies [167,168,169], morphology changes [170], and decreased capacities to mount a proper immune response have been noted [171,172].

## 6. Human Herpesvirus Type 8 (HHV-8)

HHV-8, also known as Kaposi sarcoma-associated herpesvirus (KSHV), is a double-stranded DNA virus that belongs to the gamma herpesvirus family. HHV-8 is the etiological agent of Kaposi sarcoma (KS) and other pathological conditions, including multicentric Castleman’s disease (MCD) and B-cell lymphoproliferative disorders, which can progress into KSHV-associated non-Hodgkin’s lymphoma and primary effusion lymphoma (PEL) [179]. In vitro, HHV-8 infects a broad range of cell types, including epithelial cells, B cells, dendritic cells, and monocytes, by binding to the surface receptor Siglec DC-SIGN [180,181,182,183].

### 6.1. Monocytes and TLRs

HHV-8 plays a crucial role in inducing an inflammatory cytokine milieu, particularly when the host develops a virus-associated malignancy. Chronic inflammation is a key feature of HHV-8-associated diseases. Notably, both MCD and KS-associated inflammatory cytokine syndrome (KICS) are characterized by elevated levels of IL-6 and IL-10 in the sera [184,185]. KS patients display increased proinflammatory cytokines such as IL-6 and TNF-α, as well as elevated level of the immunosuppressive cytokine, IL-10 [186]. Host et al. have shown that primary HHV-8 infection in monocytes contributes to the production of proinflammatory cytokines, such as IL-1α, IL-1β, and IL-6, which are all connected to KS development [187].

Different studies have shown HHV-8 affects monocyte functions by interfering with the TLR signaling pathway, which recognizes pathogen-associated molecular patterns (PAMPs), crucial in activating an antiviral response. In monocytes, HHV-8 infection triggers the transcription of TLR3 and its downstream targets, C-C motif chemokine ligand 2 (CCL2) and C-X-C motif chemokine 10 (CXCL10) and interferon-β (IFN-β) [188]. Conversely, in monocytes, the HHV-8 viral interferon regulatory factors (vIRFs) dampen TLR3-mediated interferon induction [189]. Another viral protein that regulates TLR signaling in HHV-8 infection is the replication and transcription activator (RTA), which promotes the proteasomal degradation of the TLR3 adaptor protein TRIF, thereby blocking the downstream signaling pathway [190]. RTA also degrades the mRNA of the adaptor myeloid differentiation primary response protein 88 (MyD88), inhibiting TLR signaling [191]. Additionally, HHV-8 infection leads to increased PD-L1 on monocytes, an inhibitory molecule that is overexpressed in many tumor types. This molecule suppresses T-cell-mediated killing and might contribute to HHV-8 immune evasion [187].

### 6.2. Monocyte Differentiation and Macrophages

HHV-8 affects monocyte survival, differentiation, and cytokine release by blocking ROS induced by macrophage colony-stimulating factor (M-CSF), preventing c-Jun N-terminal kinase (JNK) and B-cell lymphoma-2 (Bcl-2) phosphorylation and inhibiting autophagy. Additionally, HHV-8 reduces the production of proinflammatory cytokines like TNF-α and increases the release of the immune-suppressive cytokine IL-10, favoring immune suppression and viral persistence in the host [192]. In addition, recently Szymula A et al. showed that HHV-8 infection of monocytes supports viral latency in B cells. More specifically, infected monocytes and M-CSF-differentiated (M2) macrophages stimulate B-cell survival, proliferation, and differentiation into plasma blasts by releasing B-cell chemoattractant and activating factors. Furthermore, HHV-8 macrophages drive infected plasma cell differentiation and long-term viral latency [193].

Another recent study has showed that HHV-8 preferentially infects CD14+ monocytes, supporting their proliferation and differentiation into macrophages. In monocytes, the viral interleukin-6 (vIL-6) protein, a homolog of human IL-6, activates STAT1 and 3, inducing proliferation, differentiation, and proinflammatory gene expression. Additionally, vIL-6-expressing macrophages display a distinct transcriptional profile, characterized by elevated IFN-pathway activation, immune suppression, and compromised T-cell activation. These data highlight the critical role of vIL-6 in sustaining viral replication in primary monocytes by expanding infected monocytes via differentiation into longer-lived dysfunctional macrophages. This mechanism might be crucial for the virus to escape immune clearance and to support a lifelong infection [194].

VIL-6 is not the only HHV-8 protein that regulates innate host responses. The HHV-8 orfK14, which encodes for a homologous of the cellular inhibitory macrophage signal OX2 (vOX2), also affects monocyte/macrophage functions. Expression of the viral vOX2 in human primary monocyte-derived macrophages (MDMs) results in proinflammatory cytokine release and higher phagocytic activity. In contrast, an opposite effect is observed in vOX2-expressing MDMs undergoing IFN-γ activation, which results in the downregulation of cytokine production and phagocytic activity. Furthermore, vOX2-transduced MDMs display downregulation of the MHC class I and II surface molecules, which might reduce their ability to present antigens to T cells. These results highlight the immunomodulatory effects of HHV-8 vOX2 protein on monocyte biology [195].

HHV-8 infection of monocytes/macrophages is not the only way that the virus uses to impair their functions. HHV-8, by infecting endothelial cells, induces the cytokine angiopoietin-2 (Ang-2) that promotes monocyte migration. Moreover, HHV-8-infected endothelial cells produce IL-6, IL-10, and IL-13, which drive monocytes to differentiate into TAMs, by inducing TAM-specific markers such as CD163 and legumain (LGMN). Significantly, HHV-8-induced TAMs enhance tumor growth in nude mice [196].

HHV-8 primarily infects cells of the immune and vascular systems. However, HHV-8 also targets professional APCs and influences their function. Despite non-robust lytic replication, the infection affects DC immune activation, maturation, and antigen presentation. In HHV-8 infection, DCs display impaired antiviral activity. Following an immune compromising event (organ transplantation or human immunodeficiency virus type 1 (HIV) infection), a reduced HHV-8 antiviral response was noted, along with impaired DC cytokine production and antigen presentation, contributing to the development of HHV-8-associated diseases [197].

Overall, past and recent literature on the topic has shown that HHV-8 deeply impairs monocyte and macrophage functions to persist in the host and favors associated viral disease progression.

## 7. Human T-Cell Lymphotropic Virus Type 1 (HTLV-1)

Human T-cell lymphotropic virus type 1 (HTLV-1) is a complex retrovirus from the subfamily Orthoretrovirinae of Deltaretrovirus. HTLV-1 is the etiologic agent of HTLV-1-Associated Myelopathy/Tropical Spastic Paraparesis (HAM/TSP) and Adult T-cell Leukemia Lymphoma (ATLL) [198,199]. Estimates indicate that over 5 million individuals are infected with HTLV-1 in the world population. The areas with the highest prevalence are Japan; Central and South America, especially Brazil; Africa; and Melanesia [200,201]. Moreover, a related virus, HTLV-2 was discovered in 1982. It is endemic in several American Paleo-Indian populations, but population-based epidemiological studies on HTLV-1/2 are rare [202]. Most of these procedures are performed on blood donors and pregnant women. China and India, the most populous regions in the world, currently do not have reliable estimates, suggesting that the actual number of HTLV-1 carriers is unreliable [200].

In Central and South America, Africa, and Melanesia, the most prevalent HTLV-1-associated infection is HAM/TSP [200,201]. HAM/TSP is a chronic inflammatory and neurodegenerative disease characterized by demyelination and axonal degeneration in the lumbar region of the spinal cord [203]. The average evolution time between onset of neurological symptoms and paraplegia can be slow, approximately 21 years, or if it is fast, it is named subacute evolution [203,204]. Moreover, around 50% and 80% of individuals with HAM/TSP exhibit nonspecific changes in the cerebral white matter [205].

Although HTLV-1-endemic areas exit, the highest incidence of ATLL was found in southwestern Japan [206]. ALL is a CD4+ T-cell malignancy characterized by clonal integration HTLV-1 provirus. Cell activation and proliferation induce genomic instability, leading to chromosomal abnormalities that culminate in cell transformation [206,207]. ATLL can present under four clinical subtypes: acute leukemia, lymphoma, chronic, and smoldering. Some cases have been associated with cutaneous lymphomas. In addition, some of these patients had infective dermatitis, fungal or helminthic co-infections, or/and HAM/TSP [208]. Unfortunately, ATLL treatment is restricted, and patients with aggressive disease have poor prognoses due to the immunosuppression state and multidrug resistance of leukemic cells [209].

The T CD4+ lymphocytes are the principal target of HTLV-1 infection [210,211], however this virus also infects other cells, such as T CD8+ lymphocytes [212], hematopoietic stem cells [213], natural killer cells [214], dendritic cells [215], and monocytes and macrophages [216,217,218,219,220]. However, the role of innate immunity in HTLV-1 pathogenesis is unclear.

### 7.1. HTLV-1 and Monocytes

The frequency of monocytes in peripheral blood was altered in HTLV-1-infected patients [221,222], exhibiting a lower frequency of classical monocytes than uninfected individuals. In addition, Brazilian HAM/TSP patients present a lower frequency of classical monocytes [221] and a higher frequency of intermediate monocytes than uninfected individuals and asymptomatic carriers into peripheral blood mononuclear cells [221,222]. In ATLL patients, monocytes showed downregulation of CD14 and HLA-DR molecules [216]. These changes may be related to the reduction in the capacity of monocyte differentiation into dendritic cells during infection, previously demonstrated by our group [218], and as a consequence, a lower capacity for activation of T lymphocytes in ATLL and HAM/TSP, a fact already observed in HTLV-1-infected patients [216,218].

Studies suggested that hematopoietic stem cells are infected by HTLV-1, contributing to virus infection of their derivative leukocytes as monocytes and macrophages [213,223]. Monocytes and macrophages might represent the HTLV-1 reservoir in vivo. The non-classical monocytes, vasculature patrol cells, had the highest virus burden followed by classical inflammatory cells and intermediate monocytes [221,224]. It has been hypothesized that a latent infection of non-classical monocytes would allow the virus to escape immune recognition. However, monocyte infection can be impaired by SAMHD1 (SAM domain and HD domain-containing protein 1) activity. A study in vitro showed that this phenomenon occurs because of SAMHD1 apoptotic pathway activation in monocytes [225].

In monocytes and monocyte-derived dendric cells, the control of ISG gene expression involves the viral protein p30, which reduces the expression of the MxA, A3G, and OAS genes. These results suggested that protein is important to maintaining viral load because infected monocytes with p30 knockout virus produced a lower viral load [226].

In addition, monocytes have a plastic nature and pleiotropic functions, which can be modulated during a chronic infection. The label-free proteomic approach was used to characterize and compare the phenotype of monocytes derived from HTLV-1-infected patients. The results showed that chaperones, histones, and cytoskeleton proteins are differentially expressed and these effects contribute to a greater capacity for adhesion and migration of monocytes from HAM/TSP patients [219]. Using the monocytic cell line THP1, it was shown that HTLV-1 increased the levels of activation molecules HLA-DR, CD80, CD86, CD83, and chemokine receptor CCR7 related to lymphoid tissue homing [226].

In contact with infected cells, the monocytes produce inflammatory mediators, inducing TNF-α and IL-6 production [227,228]. Although HTLV-1 infection is associated with increased susceptibility to infectious diseases [229], Legionella pneumophila was rarely described. Production of TNF-α by monocytes prevented Legionella pneumophila infection of these cells in vitro [230]. Moreover, after LPS (TLR4 ligand) stimulation, infected monocytes produced higher levels of IL-1β than uninfected monocytes [227]. After R848 (TLR7/8 ligand) stimulation, the frequency of classical and non-classical monocyte producers of IL-12 was higher in HAM/TSP patients than in asymptomatic carriers. While the higher frequency of IFN-α positive cells was detected in non-classical monocytes IFN-α from HAM/TSP patients after R848 stimulation [222].

HTLV-1 infection induces the activation of CD4+ T lymphocytes, leading to spontaneous proliferation [210]. Monocytes contribute to improving T lymphocytes from HTLV-1 carrier proliferation and viral protein Tax expression in vitro [228,231].

The frequency of classical monocytes CXCR3+ and CCR5+ from HTLV-1 carriers is higher than monocytes of uninfected individuals, and in non-classical monocytes from HTLV-1 patients, CXCR3 and CCR1 are up-regulated [221]. The chemokines CCL3, CCL4, and CXCL10 and CXCL11 ligands of CCR1, CCR5, and CXCR3 were found in the cerebrospinal fluid of HTLV-1-asymptomatic carriers and HAM/TSP patients, suggesting that monocytes can migrate to the central nervous system and be involved in HAM/TSP development [232,233]. HAM/TSP patients presented a higher frequency of CX3CR1+HLA-DRhigh monocytes in circulation. Moreover, an infiltrate of CX3CR1+ CD68+ cells was identified in spinal cord tissues from HAM/TSP patients [228]. These findings suggested that monocytes were attracted to the spinal cord and differentiated into macrophages (CD68+ cells). Moreover, CD14+ monocytes from HTLV-1 individuals expressed higher levels of the adhesion molecule CD11a, which, in association with CD18, can interact with intercellular adhesion molecule 1 (ICAM-1) [232]. Soluble ICAM-1 was detected in serum and cerebrospinal fluid from HAM/TSP patients, suggesting an upregulation of this adhesion molecule in CNS. HTLV-1 infection may alter the gene expression profile of monocytes, resulting in phenotypic and functional changes, and consequently, in the differentiation and activation of macrophages.

Monocytes isolated from ATLL patients display poor ability to differentiate into monocyte-derived dendritic cells in vitro (MDDCs), with reduced antigen presentation and impaired ability to stimulate the proliferation of allogenic T lymphocytes [182,216]. On the other hand, MDDCs from HAM/TSP patients favor the proliferation of autologous CD4+ and CD8+ T lymphocytes [216]. However, their differentiation into MDDCs is also altered [216,218]. The differentiation defects of MDDCs from HAM/TSP patients are not directly due to their infection [218,228]. Impaired DC differentiation might result from an altered microenvironment in which monocytes originated. Elevated levels of IL-10 are commonly observed in ATLL patients [234]. DC development in the presence of IL-10 and TGF-β might lead to tolerance and immune evasion [228]. ATLL patients are impaired in IFN-I production [217,235] and associated with decreased DCs [217,236,237,238], suggesting a potential evasion mechanism to IFN-I antiviral control.

### 7.2. HTLV-1 and Macrophages

It is known that the Central Nervous System (CNS) contains microglia (mononuclear phagocytes important for the functioning and maintenance of the integrity of brain tissue) [239], choroid plexus macrophages, and perivascular and meningeal macrophages [240,241,242]. These cells can participate in HAM/TSP development directly. Moreover, macrophages are also found in lymphoid tissue (lymph node and spleen) where their function is associated with location (marginal zone and narrow subcapsular sinus) [243]. In lymphoid organs, macrophages are more susceptible to HTLV-1 infection, viral proteins, and cellular transformation as discussed below.

Macrophages derived from monocytic cell lines or primary monocytes can be infected in vitro after contact with HTLV-1-infected cells or a free virus [225,244,245,246]. Using monocytic cell line HTLV-1-transfected was showed that the differentiation into macrophage process can induce viral gene expression [246]; but this phenomenon was not studied ex vivo using monocytes from HTLV-1 patients.

Resident macrophages can recruit leukocytes to the infected tissues releasing chemokines. The viral protein Tax stimulation of U937 monocyte-derived macrophages induced the production and release of CCL3/MIP-1α, CCL4/MIP-1β, and CCL5/RANTES after 24 h and increasing until 21 days [247]. These findings suggested that viral protein can modulate macrophage functions and these cells can influence the cells’ arrival in HTLV-1 target tissue, such as CNS, skin, and lymphoid organs, during HAM/TSP and ATLL development.

HTLV-1-infected macrophages were found in human breast milk, and they can produce viral particles [248]. These cells can also inhibit monocyte differentiation into DCs and reduce the stimulatory capacity of presenting antigen cells to induce T-lymphocyte proliferation in vitro [249]. However, there are no studies on the role of breast milk macrophages in vertical transmission of HTLV-1.

The microbicidal activity of macrophages is important for innate immune response. The lipopolysaccharide (LPS) stimulation of macrophages derived from monocytes of HTLV-1-infected individuals induced lower levels of IL-10 than macrophages from uninfected donors. However, macrophages from HTLV-1 patients produced higher levels of CXCL9 than macrophages from uninfected individuals with or without LPS stimulation, while similar levels of CXC10 and CCL5 were detected in cultures after an LPS challenge [250].

To understand, the ATLL researchers developed transgenic mice expressing the HTLV-1 protein Tax, whose expression is sufficient to induce tumors in these animals. In 2021, Lanigan et al. demonstrated that transgenic mice expressing the HTLV-1 Tax gene and knockout for IFN-γ eveloped malignancies around the tendon sheaths and perichondrium, causing bone absorption and splenomegaly. The authors characterized a histiocytic malignancy due to F4/80 and CD11b expression and macrophage morphology of tumor cells. These malignant cells exhibited osteoclastic activity, corroborating the hypercalcemia observed in transgenic mice [251]. Similarly, ATLL patients can present hypercalcemia and bone lesions [252]. The Tax transgenic and NF-κB knockout mice developed a tumor in adrenal glands induced by macrophages Tax+. Moreover, these mice presented splenomegaly and lymphadenopathy caused by transformed macrophages [253].

In addition, HTLV-1 patients can develop granulomas. Granuloma formation is an inflammatory process common in infectious diseases in which macrophages perform an important role [254]. Macrophages can also be involved in skin granuloma origin in ATLL patients due to CD11b+ cells in the infiltrate [255,256]. Although the data suggested that macrophages are involved in mice HTLV-1 neoplasms, the relation in human disease remains unstudied.

## 8. Discussion

Accumulating evidence has shown that monocytes and macrophages are targets of viruses despite being suboptimal for viral replication. Pathogens, including viruses, attack different cell types and use diverse mechanisms to enter the cells and change their biology, promoting viral replication and spreading. Several questions remain unanswered in understanding the role of monocytes and macrophages in viral infection: (1) What is the contribution of monocytes and macrophages to the viral reservoir? (2) What is the persistence of virally infected macrophages in tissues? (3) What is the role of monocytes/macrophages in disease progression and response to therapy? (4) How can monocytes and macrophages be targeted to favor viral clearance?

Infected monocytes and macrophages, though a small immune population, have the potential to be a significant milestone in our understanding of virus-induced diseases. Future directions in the field include animal models used to study the participation of monocytes and macrophages in the development of diseases, with emphasis on neoplasms and analyses of biopsy material through large-scale single-cell sequencing techniques to characterize tumor-infiltrating human monocytes and macrophages. The findings can suggest new therapeutical approaches to modulate monocytes and macrophages during viral disease.
